# Supplementary material for: Monitoring antimicrobial resistance trends from global genomics data: amr.watch
Source: PLOS Glob Public Health. 2025 Nov 24;5(11):e0005256. doi: 10.1371/journal.pgph.0005256 (PMC12643278; doi:10.1371/journal.pgph.0005256)
Supplement: S1 Fig — The same visualisation, updated in real-time, can be viewed at: https://amr.watch/summary/all Individual visualisations for each pathogen can also be accessed from: https://amr.watch/summary. (DOCX) [file pgph.0005256.s005.docx]

**S1 Appendix**

**Supplementary Methods**

*Retrieval of WGS data and associated metadata from INSDC databases*

We retrieve all metadata associated with pathogens in the 2017 WHO priority pathogen list [1] from the European Nucleotide Archive (ENA) via the ENA Portal API on an ongoing basis. We proceed with entries (samples) from the relevant pathogens (based on the submitted classification; see **S1 Table** for accepted taxon IDs) with an available collection date from 2010 onwards that is decodable to at least the year, as well as a sampling location decodable to at least the country level. For the latter, we attempt to assign an ISO 3166-1 alpha-2 country code [2] based on information available in either the “location”, “lat”/“long” or “country” metadata fields (assessed in this order). We use the 249 “officially assigned” country codes and “other” code types where necessary (currently “XK” (Kosovo) and “AN” (Netherlands Antilles)). Reverse geocoding of latitude and longitude coordinates is performed using two open-source Node.js packages: geojson-places [3] and geojson-geometries-lookup [4] with a high quality GeoJSON map of countries' political maritime borders with 5m resolution [5]. Geocoding of location names from the “country” field is performed using the OpenCage Geocoding API [6] and the Mapbox Geocoding API [7], and samples are discarded if the resulting country codes from the two tools are inconsistent. A manually-curated list of non-standard location names and their associated country codes is also maintained and applied where necessary. Additionally, we use an additional Node.js package, country-to-iso [8], to handle inconsistent country names. Finally, collection dates (found in many different formats among the ENA metadata) are converted to a standard ISO 8601 format where they can be unambiguously interpreted. Entries that have collection dates with ranges that cover more than one year are disregarded. Only the year is extracted when the available format renders the precise date ambiguous (i.e. when the month/day values are both ≤12 and the month and day values are not equal, such as 01/02/2013).

Of those entries with a decodeable country code and collection date, we proceed with those with library_strategy=“WGS”, library_source=“GENOMIC”, library_layout=“PAIRED” and instrument_platform= “ILLUMINA”. If an entry is associated with more than one sequencing run, the run with the highest number of total bases is selected using the “base_count” field. We exclude sequencing runs that are linked to more than one entry (sample). Sequencing runs with only one fastq file are also disregarded. Sequencing runs must possess at least 20X mean coverage, based on assessing the total number of bases (“base_count”) relative to the expected length of the genome (**S1 Table**).

Sequence reads fulfilling the above criteria are downloaded from the Sequence Read Archive (SRA) using SRA-Toolkit fastq-dump v3.1.0 [9] after checking that they exist in the SRA using Entrez Programming Utilities [10].

*Genome assembly*

Sequence reads are assembled with a workflow [11] that uses the SPAdes assembler v3.15.3 [12] and which is implemented in Nextflow v21.04.1.5556 (versions as of March 2025) [13]. The workflow includes steps for quality control (QC) of the reads pre- and post-trimming, and determination of key QC metrics of the final assemblies using QUAST v5.0.2 (version as of March 2025) [14].

*Species verification*

The Speciator tool (v4.0.0) within Pathogenwatch [15] is used to verify the species of the genome assemblies. The SISTR tool (v1.1.1) [16, 17], implemented within Pathogenwatch [18], is additionally used to assign the serotype of *Salmonella enterica* genomes. Genome assemblies with a species and/or serotype identification that do not match defined taxon IDs for each pathogen (**S1 Table**) are excluded. Note that for each of *E. coli*, *S. sonnei* and *S. flexneri*, we accept genomes annotated as either *E. coli* or *Shigella* in the ENA, with the Speciator assignments used in subsequent processing (thus permitting inconsistencies in this case).

*QC of genome assemblies*

Genome assemblies that are identical to another assembly in the ongoing curated collection are excluded on the basis that these represent duplicate uploads of the same raw sequence data to INSDC databases. Assemblies are also excluded if they fail to meet one or more of our defined QC criteria, which are specific to each species/serovar (**S1 Table**). Defined thresholds for the number of contigs and N50 values were developed based on manual inspection of the species-specific distributions from all genomes assembled up to March 2022. The range of accepted GC content is the observed range among all RefSeq genomes of each species/serovar. The range of accepted assembly length values were determined using the known range among RefSeq genomes, allowing for an additional ±5%.

*Variant typing*

We use Pathogenwatch to identify the sequence type (ST) of all pathogens via available multilocus sequence typing (MLST) schemes from PubMLST [19], except for *Salmonella enterica* *subsp. enterica* serovar Typhi and *Streptococcus pneumoniae* (see below). For assemblies belonging to *Acinetobacter baumannii*, the “Pasteur” MLST scheme rather than “Oxford” scheme is used due to the presence of *gdhB* paralogues in the latter [20].

Pathogens utilising other typing schemes with amr.watch are *Salmonella enterica subsp. enterica* serovar Typhi, for which we use GenoTyphi [21] as implemented in Pathogenwatch, and *Streptococcus pneumoniae* for which we use Global Pneumococcal Sequencing Cluster (GPSC) assignments [22] as also implemented in Pathogenwatch.

*Identification of markers associated with antimicrobial resistance*

We identify genes and mutations associated with antimicrobial resistance (AMR) in each quality-controlled assembly using AMRFinderPlus v3.10.23 and database v2021-12-21 (versions as of March 2025) [23]. AMR markers are identified for antimicrobial classes defined in the 2017 WHO priority pathogen list [1], with the addition of those for quinolone resistance in *E. coli*. The list of AMR determinants included for each pathogen-antimicrobial combination is available in **S2 Table** and has been developed based on a comprehensive literature review. In particular, we disregard some markers reported by AMRFinderPlus for which there is insufficient experimental validation of their phenotypic outcome. Only complete matches to AMR markers are accepted.

**Supplementary Figures**

**
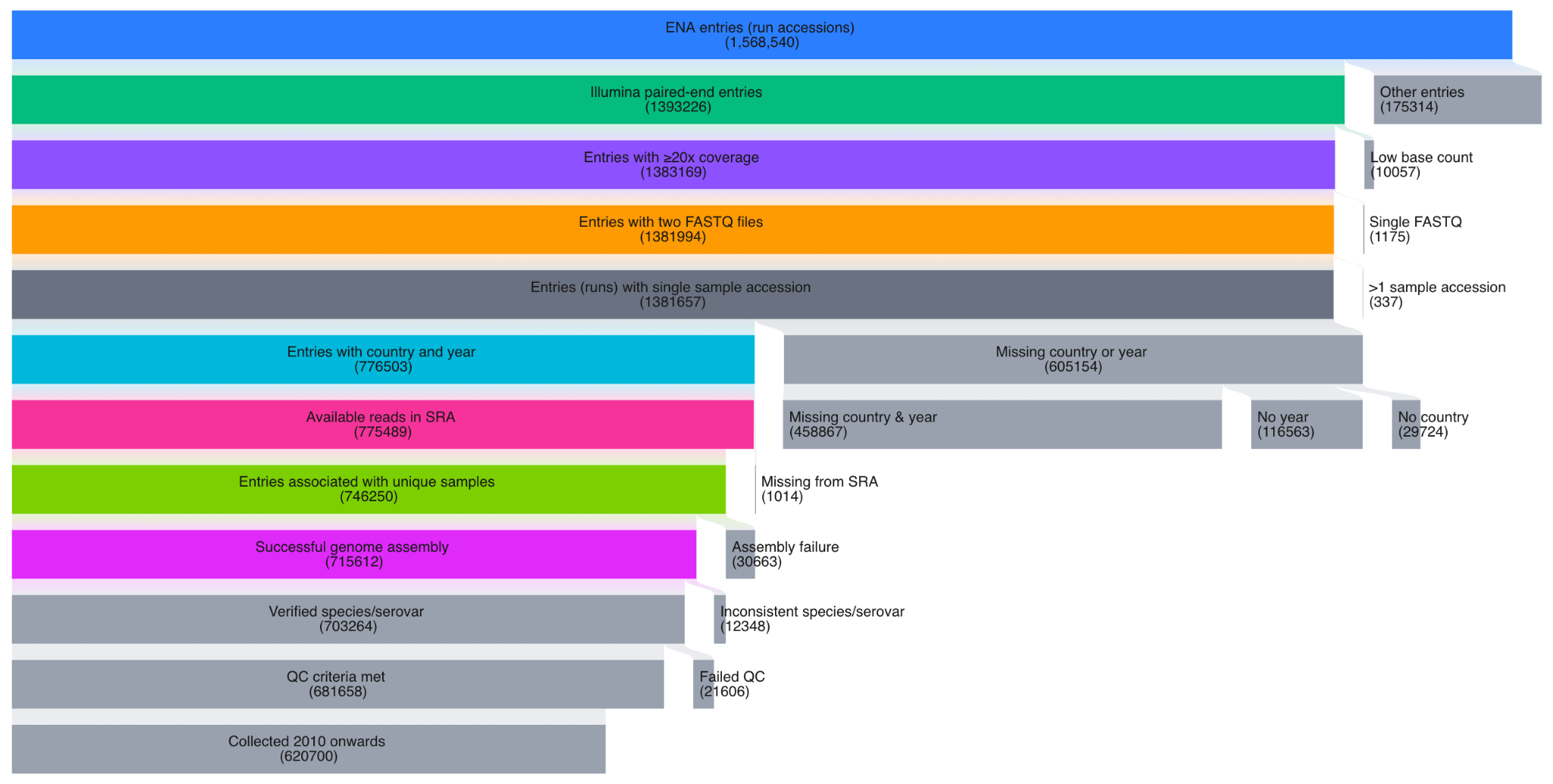
**

**S1 Fig.** Overview of the filtering process within the amr.watch workflow applied to all public genomes of priority bacterial pathogens available in the International Nucleotide Sequence Database Collaboration (INSDC) databases up to 31 March 2025. The same visualisation, updated in real-time, can be viewed at: <https://amr.watch/summary/all> Individual visualisations for each pathogen can also be accessed from: <https://amr.watch/summary>

**References**

1. World Health Organisation. Prioritization of pathogens to guide discovery, research and development of new antibiotics for drug-resistant bacterial infections, including tuberculosis. [online]. 2017 [Accessed 6 March 2025]. Available from: <https://www.who.int/publications/i/item/WHO-EMP-IAU-2017.12>
2. ISO. ISO - ISO 3166 — Country Codes. [online]. 2025 [Accessed 6 March 2025]. Available from: https://www.iso.org/iso-3166-country-codes.html
3. npm. geojson-places. (Version 1.0.8) [Software] npm, Inc. <https://www.npmjs.com/package/geojson-places>. 2023.
4. npm. Geojson-geometries-lookup. (Version 0.5.0) [Software] ndm, Inc. <https://www.npmjs.com/package/geojson-geometries-lookup>. 2020.
5. npm. geo-maps/countries-maritime-5m. (Version 0.6.0) [Software] ndm, Inc. <https://www.npmjs.com/package/@geo-maps/countries-maritime-5m>. 2018.
6. OpenCage. OpenCage Geocoding API. (Version v1) [Software] OpenCage GmbH. <https://opencagedata.com/>. 2025.
7. Mapbox. Geocoding. (Version 5) [Software] Mapbox. <https://www.mapbox.com/geocoding>. 2025.
8. Npm. country-to-iso. (Version 1.6.0) [Software] npm, Inc. <https://www.npmjs.com/package/country-to-iso>. 2025.
9. NCBI. SRA-Toolkit. (Version 3.0.0) [Software]. National Institutes of Health. <https://hpc.nih.gov/apps/sratoolkit.html>. 2024.
10. NCBI. Entrez Programming Utilities. [Online service] National Institutes of Health. <https://eutils.ncbi.nlm.nih.gov/>. 2025.
11. Centre for Genomic Pathogen Surveillance. SPAdes assembly workflow. (Version 3.15.3) [Software] University of Oxford. <https://gitlab.com/cgps/ghru/pipelines/assembly>. 2025.
12. Bankevich A, Nurk S, Antipov D, Gurevich AA, Dvorkin M, Kulikov AS, et al. SPAdes: a new genome assembly algorithm and its applications to single-cell sequencing. J Comput Biol. 2012 May;19(5):455-77.
13. Di Tommaso P, Chatzou M, Floden EW, Barja PP, Palumbo E, Notredame C. Nextflow enables reproducible computational workflows. Nat Biotechnol. 2017 Apr 11;35(4):316-319.
14. Gurevich A, Saveliev V, Vyahhi N, Tesler G. QUAST: quality assessment tool for genome assemblies. Bioinformatics. 2013 Apr 15;29(8):1072-5.
15. Centre for Genomic Pathogen Surveillance. Speciator. (Version 4.0.0) [Software] University of Oxford. <https://cgps.gitbook.io/pathogenwatch/technical-descriptions/species-assignment/speciator>. 2025.
16. Yoshida CE, Kruczkiewicz P, Laing CR, Lingohr EJ, Gannon VP, Nash JH, et al. The *Salmonella* *In Silico* Typing Resource (SISTR): An open web-accessible tool for rapidly typing and subtyping draft *Salmonella* genome assemblies. PLoS One. 2016 Jan 22;11(1):e0147101.
17. Argimón S, Yeats CA, Goater RJ, Abudahab K, Taylor B, Underwood A, et al. A global resource for genomic predictions of antimicrobial resistance and surveillance of *Salmonella Typhi* at pathogenwatch. Nat Commun. 2021 May 17;12(1):2879.
18. Centre for Genomic Pathogen Surveillance. SISTR (Pathogenwatch). (Version 1.1.1) [Software] University of Oxford. <https://cgps.gitbook.io/pathogenwatch/technical-descriptions/typing-methods/sistr>. 2024.
19. Jolley KA, Bray JE, Maiden MCJ. Open-access bacterial population genomics: BIGSdb software, the PubMLST.org website and their applications. Wellcome Open Res. 2018 Sep 24;3:124.
20. Gaiarsa S, Batisti Biffignandi G, Esposito EP, Castelli M, Jolley KA, Brisse S, et al. Comparative analysis of the two *Acinetobacter baumannii* multilocus sequence typing (MLST) schemes. Front Microbiol. 2019 May 3;10:930.
21. Dyson ZA, Holt KE. Five years of GenoTyphi: Updates to the global *Salmonella* Typhi genotyping framework. J Infect Dis. 2021 Dec 20;224(12 Suppl 2):S775-S780.
22. Gladstone RA, Lo SW, Lees JA, Croucher NJ, van Tonder AJ, Corander J, et al. Global Pneumococcal Sequencing Consortium. International genomic definition of pneumococcal lineages, to contextualise disease, antibiotic resistance and vaccine impact. EBioMedicine. 2019 May;43:338-346.
23. Feldgarden M, Brover V, Gonzalez-Escalona N, Frye JG, Haendiges J, Haft DH, et al. AMRFinderPlus and the Reference Gene Catalog facilitate examination of the genomic links among antimicrobial resistance, stress response, and virulence. Sci Rep. 2021 Jun 16;11(1):12728.
