# Supplementary material for: Monitoring antimicrobial resistance trends from global genomics data: amr.watch
Source: PLOS Glob Public Health. 2025 Nov 24;5(11):e0005256. doi: 10.1371/journal.pgph.0005256 (PMC12643278; doi:10.1371/journal.pgph.0005256)
Supplement: Attachment — Submitted filename: Response to Reviewers.docx [file pgph.0005256.s007.docx]

Dear Hui-min,

Thank you for the reviews and below we past the reviewers comments and respond to each (in red text). We very much appreciate all your efforts and support through the process.

All the very best

David Aanensen (on behalf of co-authors)

**Reviewer #1**: This manuscript introduces amr.watch, an interactive web-based platform designed to leverage public whole genome sequencing (WGS) data to monitor global trends in antimicrobial resistance (AMR). The platform currently integrates 620,700 high-quality genomes from WHO-priority bacterial pathogens with geotemporal metadata. The manuscript presents the platform's methodology, data coverage, analytical capabilities, and potential public health utility.

The Strengths of the study include the development of a transparent, scalable, and user-friendly tool for AMR trend analysis, the strict genome curation, species specific AMR mechanisms and open access / reusability of the data.

There are clear weaknesses which are acknowledged by the authors, in particular, significant data biases, especially underrepresentation from LMICs and the selection criteria for sequenced pathogens (eg. foodborne diseases, outbreaks); limited metadata; lack of formal validation studies comparing genomic predictions to phenotypic resistance data.

RESPONSE: We thank the reviewer for their comments and address each below:

There are a number of areas to address before publication.

Specific recommendations
1. Although the authors acknowledge metadata incompleteness, this should be highlighted earlier (e.g., in Abstract and Introduction) with specific examples of how it may bias findings.

RESPONSE: We have added text to indicate that our initial focus is on those genomes deposited in public archives which are firstly, of high quality, and secondly have allied spatial and temporal metadata. We indicate that additional metadata is problematic in current archives and indicate that the enhanced collection of allied metadata is crucial for more robust conclusions and is an area of focus in ensuring archival data becomes increasingly more useful within public health decision making.

2. A table summarizing the metadata fields available and their completeness across pathogen groups would improve transparency.

RESPONSE: We thank the reviewer for this comment and have made reference to incompleteness in both the abstract and introduction.  We purposely took the stance to only focus on four axes of information within our collective approach, and this was based on approaches taken within the pandemic around SARS-CoV-2 genomes.  The four axes being time / place (as the metadata) along with genotype (variant or in the case of bacterial species within amr.watch - MLST or equivalent strain nomenclature) and risk (in this case the genetic ‘determinant’ of resistance, based on the WHO priority list).  We describe that these four pieces of information alone enabled ‘us’ globally to monitor the emergence, spread and handling of COVID variants during the pandemic and that this paradigm fits for AMR.    We purposely didn’t validate all metadata fields as they are very incomplete across genome data - our aim is to summarise this information subsequently and to address disparity. (also linking  to metadata incompleteness - the amr.watch overview <https://amr.watch/summary> indicates numbers of genomes which do and don’t have the full set of data).

3. Include a benchmarking subsection comparing genotypic AMR calls in amr.watch to matched phenotypic data (where available) or at least discuss its feasibility and limitations.

RESPONSE: We have purposely focussed Amr.watch on genomic data at this time as the public archives (INSDC databases) do not require the submission of phenotypic data and therefore there is a vast lack of data beyond that of the other meta data above.  Clearly this is a situation that requires change and we hope that our approach support efforts to co-ordinate the genomic data in ways that lead to increased metadata deposition. There are endeavours to alleviate this (including amrRUles and the WHO led cataloguing exercises ) and we expect phenotype / genotype requirements to mature in the next years - We have a section in Discussion which describes the need and lack of data and have expanded this to explicitly describe the need for matched phenotypic data.

4. Provide more structured detail (e.g., heatmaps) on country-level contributions by income group, to visually emphasize the imbalance.

RESPONSE: We thank the reviewer for the suggestion , however the colour schemes included already follow the heatmap structure - due to the vast disparity between HIC’s (and in particular the US and UK) the gradations of colour and boundaries within traditional heatmaps would essential end up only having 2 colours (ie very hot and very cold).   We have included within the ‘Overview’ section of the site (available from the front page), the ability to show availability of data grouped by world bank income group, which can either be viewed by all data or, by selecting a particular species (or priority group) be viewed as a heatmap (purple to white) showing distribution.   It’s very clear that all categories show the same general disparity and we hope that highlighting in this very simple format will support efforts.

5. While the authors discuss ambitions to increase global representation, suggesting specific actions for global surveillance equity, such as collaborations, data sharing incentives, or funding strategies would be helpful.

RESPONSE: The discussion includes a section on describing and calling for global efforts of collaboration and funding to forward these aims.

6. While the utility for researchers and policymakers is stated, more specific examples would help. How are ministries of health expected to use this tool in real time? Could it support AMR forecasting, outbreak alerts, or antimicrobial stewardship? This is important to demonstrate practical utility of the platform.

RESPONSE: The discussion includes a description of potential utility of the platform and aims for further utility however we have expanded the last paragraph in the discussion to be more explicit.

7. Given point 6 above, the authors should undertake an evaluation of utility of the platform with “end users” (to ensure the data / presentation) has the utility as stated by the authors. This could/should include a combination of academics and policy makers.

RESPONSE: The application has been developed in conjunction with the NIHR global health research Unit of genomic surveillance of AMR, including public health experts (from national agencies) and policy makers (eg from ministries of health - specifically in the Philippines), along with academics, engineers and UX/UI experts within the field of Human-Computer interactions and web technology and design.    We have a history of producing web applications that have end user utility built into the initial design process, from a stakeholder perspective and we spent considerable time liaising with end users to assess initial representation.

It is is very clear (and this is based on 15 years of application development of toolsets like [Microreact.org](http://microreact.org) / pathogen.watch, [five.epicollect.net](http://five.epicollect.net) etc) that iterative design, UX/UI and feedback <-> engineering process leads to the most useable and valuable toolsets and resources. Stakeholder-centric design is at the forefront of our translational work - We follow these design and architectural processes and will continue to iterate functionality and assess utility on these basis.  In addition, we have presented amr.watch at various conferences (both academic and public health) and more formally to the WHO for critical feedback that has led to a redesign and the current iteration.

**Reviewer #2**: This paper presents a new web application, amr.watch—a platform that integrates, analyses, and visualises whole genome sequencing (WGS) data of WHO-defined priority bacterial pathogens. The scope and fluid functionality of this “always-on” web application make it one of the first of its kind to provide public access to geotemporal trends in antibacterial pathogen genomes. The development of this tool is undoubtedly significant in supporting global antimicrobial resistance surveillance.

RESPONSE: We thank reviewer 2 for thoughtful and insightful comments and acknowledgement of the significant impact that our approaches may make.

Minor comments:
1. Line 172 – Only Illumina short-read sequences are currently made accessible in amr.watch. It would be beneficial to address the exclusion of long-read sequences (Nanopore/PacBio) deposited in INSDC, especially considering the growing prominence of nanopore sequencing in the diagnostics field. A brief discussion on this point would provide valuable context.

RESPONSE: We have included in the methods section a point regarding Long read vs short read data

2. Line 185 – It is advisable to briefly describe how Speciator assigns taxonomy to genomes. A concise explanation (one to two sentences) would enhance clarity for readers unfamiliar with the tool.

RESPONSE: We have included a link to the speciation documentation within the methods secretion which describes how assignments are produced - <https://tinyurl.com/speciator>

**Reviewer #3**: This manuscript presents a novel web platform, “amr.watch,” which allows users to visually and intuitively explore the geographical and temporal trends of sequence types (STs) and major AMR determinants across 16 bacterial species. The platform incorporates data from 620,700 pathogen genomes with geotemporal metadata, with variable representation of different species and geographic regions. This is an innovative and valuable tool. However, I have several major comments, as outlined below:
________________________________________
1.
"AMR mechanisms count over time" window: This visualization should allow stratification by each individual AMR determinant. For example, in the case of S. pneumoniae, the “AMR mechanisms overview” window displays counts for each of the three pbp genes separately, whereas the “AMR mechanisms count over time” window shows only the combined number of genomes carrying any of the three. It should instead allow separate visualization for each pbp gene.

RESPONSE: thank you to the reviewer for comment – however, this is actually achievable by, when viewing ‘AMR mechanisms count over time’ selecting a specific mechanism (eg clicking the pbp gene of choice) which will filter the mechanism count over time accordingly (as in screenshot below)

We thank the reviewer for the comment and will update the documentation to make this function clearer.
________________________________________
2.
On the data summary page (<https://amr.watch/summary>), the date when the data were tabulated should be explicitly stated. Additionally, the frequency of genome data updates in amr.watch should be clarified.

RESPONSE: The data summary page - available here : <https://amr.watch/summary> contains dates for when (for each species) the data have been updated - this reflects the real-time ‘always-on’ ingest of data (and QC etc as described).
________________________________________
3.
I recommend clarifying how the data coverage in amr.watch compares with that of the allthebacteria project (<https://allthebacteria.readthedocs.io/en/latest/>), since both collect data extensively from public databases (e.g., SRA/ENA). For instance, amr.watch shows fewer than 50 S. aureus genomes per year in the UK since 2020, whereas the allthebacteria project includes 169 isolates from the UK in 2022 alone.

RESPONSE:  we have purposely focussed on High quality genomic assemblies (methods) and those that both contain only time AND place within the accompanying metadata - allthebacteria has a somewhat different focus, including all genome data (both HQ and others, and also any genome with now or partial time/space metadata)  the difference in numbers is reflected here.   We firmly believe that the public health focus within amr.watch shoul be addressed towards high quality data only, along with the (initially) very minimal time/place combination of data to highlight, firstly the lack of controlled data and also point to wards value for public health going forward.

________________________________________
4.
For the CSV file downloadable from the amr.watch website, it would be valuable to include the data source or reference for each isolate, if possible.

RESPONSE: ENA Accession numbers are present within the CSV download which links to the full ENA entry for each record.

________________________________________
5.
In the “Variants overview” window, it would be helpful if the authors could include single- or double-locus variants of each ST when the percentage of such variants is not negligible.

RESPONSE : we thank the reviewer and this is an insightful suggestion. We feel though that for initial release this may lead to confusion, but are looking into how to incorporate for release cycle.  We also feel that relationships between (types) will become increasingly important - certainly for the more clonal organisms.

________________________________________
6.
The data displayed for each bacterial species sometimes contain errors or require improvement, as detailed below:
• E. coli: ST is always displayed as "null," which appears to be a bug in the system. Additionally, when clicking on an AMR determinant in the “AMR mechanism overview” window, the y-axis of the “AMR mechanism count over time” window shows inappropriate “%” marks (e.g., 500%), which should be corrected.

RESPONSE:  fixed

• E. faecium: “Novel” MLST appears as the third most frequent in the “Variants overview” window. However, when I downloaded data of isolate ERR10431341 using fastq-dump, it was identified as ST1424 rather than novel, after assembly and MLST typing. Additionally, since daptomycin and linezolid are essential antimicrobials for treating Enterococci, resistance determinants for these drugs should be included.

RESPONSE: Thank you for pointing out - we have updated the scheme

• Campylobacter: As macrolides are the first-line treatment, resistance determinants for macrolides should be added.

• N. gonorrhoeae: For cephalosporin resistance, mosaic penA alleles are more critical than single amino acid substitutions. These should be included, and can be typed using the pyngoST tool.

RESPONSE: We have brokered a collaboration with the pyngoST developers for further addition.

• S. Typhi: ST is always shown as "null," which likely reflects a bug.

RESPONSE: fixed

• S. aureus: The “AMR mechanisms overview” shows one VRSA isolate, but the “AMR mechanism count over time” does not. Is there any true record of VRSA that was correctly validated? Additionally, since daptomycin is also an essential antimicrobial for treating S. aureus, resistance determinants for it should be included.

RESPONSE: We have focussed on the WHO priority drugs, however have included MecC2 within the display settings.  We will aim to address the issue within the presence of vancomycin VanA vs full operon through alignment with community best practice.

• H. influenzae: The proportion of “Others” in the ST distribution appears excessively high and should be reviewed.

RESPONSE: Thank you to the reviewer - it’s quite clear that the high diversity of H. influenzae genotypes is a consequence of the high diversity generally within the species (https://onlinelibrary.wiley.com/doi/full/10.1002/jcla.23145) and as a consequence highlights the need for focussed approaches on whether our current typing methods are adequate. We feel addressing this is outside of the scope of the manuscript, but agree that it should be addressed within the community. One thing we hope is that presenting the global data in this way will lead to enhancing our collective discussions on the ongoing use of WGS on a species by species level, along with the development of more refined typing / genotyping methods - we have seen this enhanced with WGS for S. pneumoniae (eg GPSCs ) and S. typhi (genotyphi).
